# INNOVATIVE PERSON-CENTERED SERVICES THROUGH THE KŪPUNA SUPPORT NAVIGATORS

**DOI:** 10.1093/geroni/igae098.3859

**Published:** 2024-12-31

**Authors:** Jenny Jinyoung Lee, Christy Nishita

**Affiliations:** University of Hawaii at Manoa, Honolulu, Hawaii, United States; University of Hawaii, Honolulu, Hawaii, United States

## Abstract

The Kūpuna Support Navigator Program in Hawai’i employs Community Health Workers, known as Kūpuna Support Navigators, to connect kūpuna (older adults in Hawaiian) with essential community resources tailored to their specific needs. This innovative, person-centered approach significantly enhances the aging support network in Hawai’i through comprehensive education, seamless integration of care services with advanced tracking software, and rigorous impact evaluation. Its strategic design offers scalable, replicable, and sustainable solutions that enhance the ability of older adults across diverse communities to age-in-place. This program increases the availability of trained Community Health Workers embedded in the aging support network, fostering a supportive environment that enhances the well-being and independence of older adults, ensuring they receive better support and care. The program encompasses a holistic framework that addresses immediate health and social needs while focusing on long-term sustainability and community empowerment. In this presentation, we will detail the setup, structure, and processes of the Kūpuna Support Navigator Program. We will leverage data visualization to present specific outcomes that illustrate the program’s effectiveness in meeting the complex needs of its target population. Additionally, we will discuss the lessons learned and best practices developed, emphasizing the program’s potential as a model for similar initiatives. This overview aims to highlight the significant role of the Kūpuna Support Navigator Program in promoting and enhancing the well-being and independence of older adults.

